# The Treatment of Intussusception

**Published:** 1901-10

**Authors:** 


### The Treatment of Intussusception.

The September 15th number of *Pediatrics* contains a paper on the treatment of intussusception as read before the British Medical Association by Bernard Pitt, F. R. C. S. With reference to injection the author says: "The most unsatisfactory feature of injection is that one is never certain that success is complete. In operating upon infants one is struck by the difficulty in effecting complete reduction even when manipulating the bowel outside of the abdomen. It is often difficult to say whether the thickening present is due to the incomplete reduction, to oedema, or to some other conditions, such as polypus within the bowel."

He would reserve inflation or injection for cases seen within a few hours. Shock must be minimized by warmth and by rapidity of operation.

He opens the abdomen in the middle line and incises the distended bowel to allow the air and contents to escape.

A common accident is tearing of the peritoneal coat of the ensheathing bowel, therefore, gentle manipulation is necessary.

To lessen the danger of ventral hernia he uses both buried and superficial sutures.

